# Tau Aggregation‐Dependent Lipid Peroxide Accumulation Driven by the hsa_circ_0001546/14‐3‐3/CAMK2D/Tau Complex Inhibits Epithelial Ovarian Cancer Peritoneal Metastasis

**DOI:** 10.1002/advs.202310134

**Published:** 2024-04-18

**Authors:** BinShu Chai, Yong Wu, HengHui Yang, BiaoFeng Fan, SiYu Cao, XiaoFei Zhang, YaQing Xie, ZhiXiang Hu, ZhongLiang Ma, YunKui Zhang, Wei Pan, Wei Meng, Jiao Meng, WenJuan Tian, JiaLi Zhang, YanLi Li, Yang Shao, ShaoJia Wang

**Affiliations:** ^1^ Department of Gynecology The Third Affiliated Hospital of Kunming Medical University Yunnan Cancer Hospital Yunnan Cancer Center Kunming 650118 China; ^2^ Lab for Noncoding RNA & Cancer School of Life Sciences Shanghai University Shanghai 200444 China; ^3^ Department of Oncology Shanghai Medical College Fudan University Shanghai 200032 China; ^4^ Department of Gynecologic Oncology Fudan University Shanghai Cancer Center Shanghai 200032 China; ^5^ Department of Gynecology Shanghai First Maternity and Infant Hospital Tongji University School of Medicine 2699 West Gaoke Road Shanghai 201204 China; ^6^ Department of Integrative Oncology Fudan University Shanghai Cancer Center and Institutes of Biomedical Sciences Fudan University Shanghai 200032 China; ^7^ Department of Anesthesiology Fudan University Shanghai Cancer Center Shanghai 200032 China; ^8^ Cancer Institute Fudan University Shanghai Cancer Center, and Shanghai Fifth People's Hospital Shanghai 200032 China

**Keywords:** 14‐3‐3, epithelial ovarian cancer, ferroptosis, lipid peroxide, Tau

## Abstract

Intraperitoneal dissemination is the main method of epithelial ovarian cancer (EOC) metastasis, which is related to poor prognosis and a high recurrence rate. Circular RNAs (circRNAs) are a novel class of endogenous RNAs with covalently closed loop structures that are implicated in the regulation of tumor development. In this study, hsa_circ_0001546 is downregulated in EOC primary and metastatic tissues vs. control tissues and this phenotype has a favorable effect on EOC OS and DFS. hsa_circ_0001546 can directly bind with 14‐3‐3 proteins to act as a chaperone molecule and has a limited positive effect on 14‐3‐3 protein stability. This complex recruits CAMK2D to induce the Ser324 phosphorylation of Tau proteins, changing the phosphorylation status of Tau bound to 14‐3‐3 and ultimately forming the hsa_circ_0001546/14‐3‐3/CAMK2D/Tau complex. The existence of this complex stimulates the production of Tau aggregation, which then induces the accumulation of lipid peroxides (LPOs) and causes LPO‐dependent ferroptosis. In vivo, treatment with ferrostatin‐1 and TRx0237 rescued the inhibitory effect of hsa_circ_0001546 on EOC cell spreading. Therefore, based on this results, ferroptosis caused by Tau aggregation occurs in EOC cells, which is not only in Alzheimer's disease‐ or Parkinson's disease‐related cells and this kind of ferroptosis driven by the hsa_circ_0001546/14‐3‐3/CAMK2D/Tau complex is LPO‐dependent rather than GPX4‐dependent is hypothesized.

## Graphical abstract

hsa_circ_0001546 fulfills the14‐3‐3/Tau role in maintaining ferroptosis. hsa_circ_0001546 stabilizes 14‐3‐3 proteins and acts as their chaperone molecule. The hsa_circ_0001546/14‐3‐3/CAMK2D/p‐Tau complex drives Tau aggregation via Ser324 phosphorylation of Tau in EOC cells and induces LPO‐dependent ferroptosis to inhibit EOC spread and metastasis.

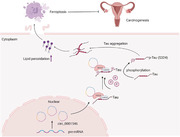

## 1 Introduction

As the most lethal gynecological malignancy, epithelial ovarian cancer (EOC) accounts for 70%–80% of all ovarian cancer (OC) cases and has a 5‐year survival rate of at most 45%, and the rate is sometimes much lower.^[^ ^1^ ^]^ In terms of clinical advancement, the metastasis of EOC occurs mainly through intraperitoneal dissemination and lymphatic metastasis and thus induces the occurrence of omentum and peritoneum metastasis.^[^ ^2^ ^]^ Therefore, exploring previously unidentified mechanisms of EOC metastasis may provide a novel strategy for EOC clinical treatment.

Ferroptosis is a kind of novel programmed cell death driven by iron‐dependent phospholipid peroxidation^[^ ^3^ ^]^ and was found to be closely related to cancers in the early stages of cancer research.^[^ ^4^ ^]^ The 2 main molecular mechanisms of ferroptosis include lipid peroxidation and the canonical GPX4‐regulated ferroptosis pathway, and the accumulation of lipid peroxides (LPOs) is the hallmark of ferroptosis.^[^ ^5^ ^]^ Microtubule‐associated protein Tau (MAPT) has been found to be correlated with several neurodegenerative disorders, such as Alzheimer's disease (AD).^[^ ^6^ ^]^ Tau aggregation, induced by abnormal phosphorylation of Tau, has been verified as one of the main causes of AD. In addition, according to a limited number of studies, Tau may play different roles in cancers. In IDH1‐mutant diffuse glioma, Tau was upregulated; phenotype, which was driven by mutant IDH1 and impeded neovascularity, was associated with a better prognosis.^[^ ^7^ ^]^ In EOC, both the downregulation and overexpression of the Tau protein could inhibit cell proliferation, and Tau acted as a marker of paclitaxel sensitivity in EOC.^[^ ^8^ ^]^ Tau protein is also considered linked with ferroptosis. Abnormal phosphorylated and aggregated Tau protein can cause iron accumulation, oxidative stress, and lipid peroxidation and finally result in ferroptosis to promote memory impairment induced by Alzheimer's disease.^[^ ^9^ ^]^ Besides, tau protein misfolding was found to be related to iron accumulation in Alzheimer's disease, which caused oxidative stress, neuron loss, and ferroptosis.^[^ ^10^ ^]^ Although the relationship between Tau and ferroptosis has been illustrated in neurodegenerative diseases, there is still a lack of relevant studies in cancers.

The 14‐3‐3 family of proteins (tyrosine 3‐monooxygenase/tryptophan 5‐monooxygenase activation protein, YWHA) includes 7 main isoforms, and these proteins act as adaptors or chaperone molecules in eukaryotic cells.^[^ ^11^ ^]^ By interacting with other proteins, 14‐3‐3 proteins can form homodimers or heterodimers.^[^ ^12^ ^]^ Among these proteins, more than one hundred interact with 14‐3‐3 in a phosphorylation‐dependent manner, including protein kinases, receptor proteins, and enzymes.^[^ ^13^ ^]^ 14‐3‐3 proteins are abundant in the central nervous system, and evidence shows that 14‐3‐3 proteins are upregulated in Alzheimer's disease and Parkinson's disease (PD).^[^ ^14^ ^]^ The interaction between 14‐3‐3 and Tau is the research hotspot of neurodegenerative disorders, and 14‐3‐3ζ and 14‐3‐3σ were successively found to stimulate in Tau aggregation.^[^ ^15^ ^]^ Although this close relationship between 14‐3‐3 and Tau has received sustained attention in nervous system research, related studies in cancers are still lacking.

Unlike any other type of linear noncoding RNAs (ncRNAs), circular RNAs are covalently closed single‐stranded ncRNAs with a back‐splicing junction site (BSJ).^[^ ^16^ ^]^ In various studies, circRNAs have been found to have many cellular roles, including modulating transcription, interacting with mRNAs, sponging miRNAs, and producing translation products.^[^ ^17^ ^]^ In view of their stability and broad functions in eukaryotic cells, circRNAs are thought to be a new generation of targeted therapies for cancer.^[^ ^18^ ^]^

In this study, we found some significantly up‐ or downregulated circRNAs based on RNA‐Seq data of 8 normal ovarian tissues and 10 ovarian cancer (OC) tissues and verified the downregulation of hsa_circ_0001546 in OC. In vitro and in vivo assays confirmed the inhibitory action of hsa_circ_0001546 on OC cell proliferation and migration. Regarding the molecular mechanism, hsa_circ_0001546 acts as the chaperone molecule of the 14‐3‐3 protein, which supports the molecular chaperone function of 14‐3‐3 protein. By directly binding to 14‐3‐3, the hsa_circ_0001546/14‐3‐3 complex can recruit calcium/calmodulin‐dependent protein kinase II delta (CAMK2D) to induce abnormal phosphorylation of the Tau protein at the Ser324 site. Phosphorylation of Tau bound to 14‐3‐3 causes Tau aggregation‐dependent LPO accumulation and ferroptosis, which ultimately blocks OC progression.

## 2 Results

### 2.1 hsa_circ_0001546 is Gradually Downregulated in Paired Primary Ovarian Cancer, Omentum Metastasis, and Peritoneum Metastasis Tissues

**Figure 1:**
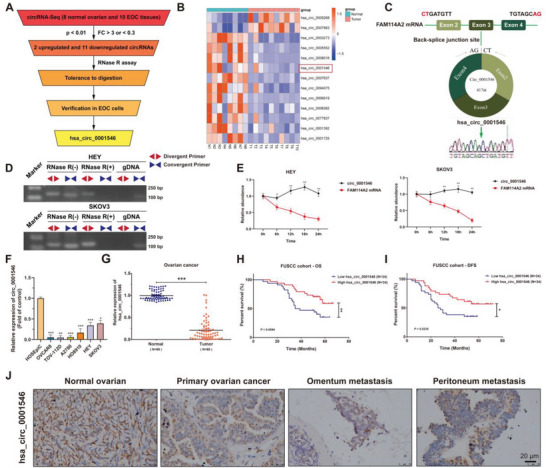
hsa_circ_0001546 is identified as a circular RNA and downregulated in EOC tissues. A) The flowchart illustrates the selection processes of hsa_circ_0001546 based on RNA‐seq data. B) The hierarchical clustering heatmap shows the most differentially expressed circRNAs in normal ovarian tissues and EOC tissues (Fold change, FC > 3 and < 0.3, *p*‐value < 0.01). Red in the heatmap represents upregulation. Blue represents downregulation. C) The ideograph illustrates the genomic location and splicing mode of hsa_circ_0001546. The splicing junction site was confirmed by Sanger sequencing. D) The RNase R digestion test was performed to validate the stability of hsa_circ_0001546 in HEY and SKOV3 cells. E) Analysis for RNA abundance of hsa_circ_0001546 and FAM114A2 treated with Actinomycin D (2 µg/mL) at the indicated time point. F) The expression of hsa_circ_0001546 in normal control HOSEpiC cell and EOC cells including OVCAR8, TOV‐112D, A2780, HO8910, HEY, SKOV3 was detected by RT‐qPCR assays. G) hsa_circ_0001546 expression was tested in 69 human normal ovarian tissues (normal, *n* = 69) and 68 EOC tissues (tumor, *n* = 68) using RT‐qPCR. H) The overall survival rate (OS) analysis of hsa_circ_0001546 in FUSCC cohort. I) The disease‐free survival rate (DFS) analysis of hsa_circ_0001546 in FUSCC cohort. J) The RNA level of circ_0001546 was detected in normal ovarian tissues and paired primary ovarian cancer, omentum metastasis, and peritoneum metastasis tissues using ISH. Scale bar = 20 µm. ^*^ *p* < 0.05, ^**^ *p* < 0.01, ^***^ *p* < 0.001. Three independent experiments were performed.

RNA sequencing on 8 normal ovarian tissues and 10 EOC tissues was carried out, and differentially expressed circRNAs were screened out (fold change, FC > 3 and <0.3, *p* value < 0.01). A total of 13 dysregulated circRNAs were identified in EOC tissues; of these circRNAs, 2 were upregulated and 11 were downregulated. The screening results were verified by RT‒qPCR using specific primers, and we finally focused on investigating hsa_circ_0001546, whose expression was significantly decreased in EOC tissues (**Figure** **1**A,B).

hsa_circ_0001546 is generated from exons 2–4 of the FAM114A2 gene located on human chromosome (chr) 5 and has a length of 417 nt. The back‐splice junction (BSJ) site of hsa_circ_0001546 was amplified using divergent primers and confirmed by Sanger sequencing (Figure 1C). The sequence “AGCT” of the BSJ site is consistent with circBase database annotation (http://www.circbase.org/). To test its stability, the total RNA was divided into 2 groups before PCR testing. One was pretreated with RNase R, and the other was used as a control without RNase R treatment. The convergent primer to amplify FAM114A2 mRNA and divergent primer to amplify hsa_circ_0001546 were designed. The results showed that FAM114A2 mRNA was notably decreased after RNase R treatment, while hsa_circ_0001546 was resistant to RNase R digestion. In addition, hsa_circ_0001546 could only be amplified by divergent primers in cDNA rather than genomic DNA (gDNA), which supported the formation of hsa_circ_0001546 by post‐transcriptional back‐splicing (Figure 1D). We further determined the stability of hsa_circ_0001546 by actinomycin D (Act D) treatment. The results demonstrated that hsa_circ_0001546 was more stable than FAM114A2 mRNA (Figure 1E). These results demonstrate that hsa_circ_0001546 is a kind of stable circRNA. To further verify the results of the above RNA sequencing, we detected the expression of hsa_circ_0001546 in various common EOC cell lines. Consistent with the above results, the expression level of hsa_circ_0001546 was also significantly downregulated in EOC cell lines in comparison to HOSEpiC, a human ovarian surface epithelial cell line (Figure 1F). In 69 normal ovarian tissues and 68 EOC tissues, the RNA level of circ_0001546 was found to be lower in EOC tissues than in normal ovarian tissues (Figure 1G). Based on the median value, 68 EOC samples (FUSCC cohort) were divided into 2 groups (low and high hsa_circ_0001546 expression groups). The results showed that high hsa_circ_0001546 levels were associated with both better overall survival and better disease‐free survival in the FUSCC cohort (Figure 1H,I). In addition, we detected the expression of hsa_circ_0001546 in paired normal ovarian tissues, primary ovarian cancer, omentum metastasis tissues, and peritoneum metastasis tissues using ISH and verified that hsa_circ_0001546 was positively downregulated in EOC tissues (Figure 1J). These results indicate that circ_0001546 is frequently decreased in EOC and negatively correlates with EOC malignant features.

### 2.2 hsa_circ_0001546 is Mainly Localized in the Cytosol of Cells and Inhibits EOC Progression In Vitro

**Figure 2:**
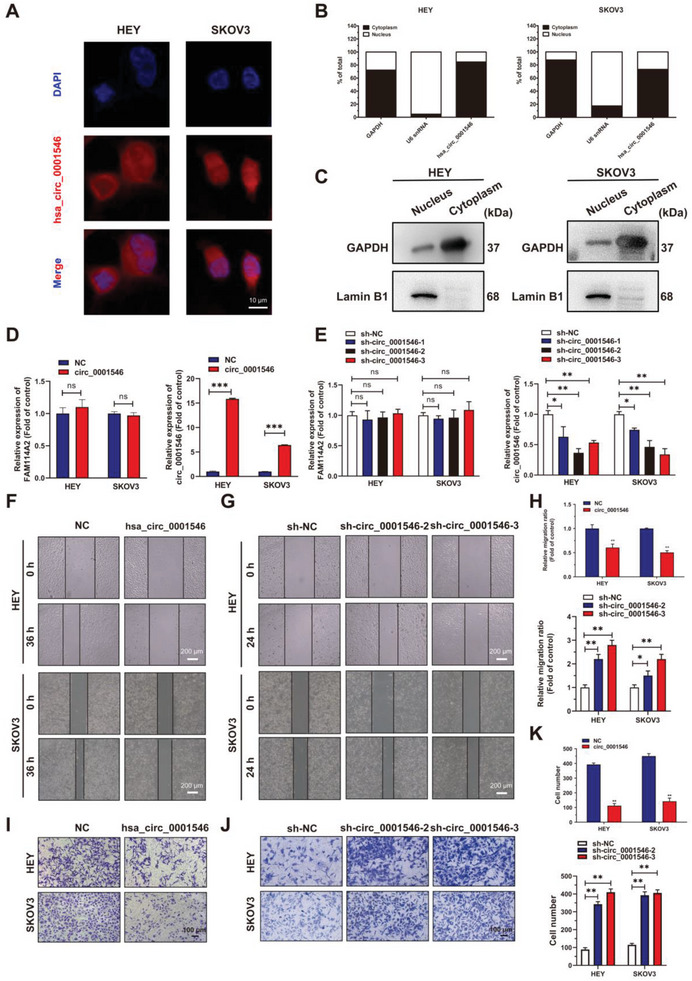
hsa_circ_0001546 is mainly localized in the cytosol of EOC cells and inhibits EOC metastasis in vitro. A) Subcellular localization of hsa_circ_0001546 detected by FISH assay in HEY and SKOV3 cells. Scale bar = 20 µm. B) HEY and SKOV3 cells were subjected to cellular fractionation followed by RNA extraction and RT‐qPCR analysis to quantify the amount of hsa_circ_0001546 in both the nucleus and cytosol of the cells. GAPDH and U6 snRNA served as purity controls for cytosolic and nuclear fractions, respectively. C) The quality of the cellular fractionation assay was detected using western blot. GAPDH and Lamin B1 served as purity control for cytosolic and nuclear fractions, respectively D) The expression of hsa_circ_0001546 and FAM114A2 mRNA in HEY and SKOV3 was detected using RT‐qPCR after transfected with a hsa_circ_0001546 overexpressing vector. E) The expression of hsa_circ_0001546 and FAM114A2 mRNA in HEY and SKOV3 was detected using RT‐qPCR after transfected with the hsa_circ_0001546 knockdown vectors. F–H) Wound healing assay for HEY and SKOV3 cells with hsa_circ_0001546 overexpression or knockdown. Scale bar = 200 µm. I–K) Transwell assay for HEY and SKOV3 cells with hsa_circ_0001546 overexpression or knockdown. Scale bar = 100 µm. ^*^ *p* < 0.05, ^**^ *p* < 0.01, ^***^ *p* < 0.001. Three independent experiments were performed.

FISH and cellular fractionation assays revealed that hsa_circ_0001546 was predominantly localized in the cytoplasm (**Figure** **2**A–C). To investigate the role of hsa_circ_0001546 in EOC progression, we upregulated hsa_circ_0001546 expression using a hsa_circ_0001546 overexpression vector and knocked down hsa_circ_0001546 expression in EOC cells using shRNAs specifically targeting the BSJ site of hsa_circ_0001546 without affecting the level of FAM114A2 mRNA (Figure 2D,E). Subsequently, CCK‐8 and colony formation assays showed that hsa_circ_0001546 overexpression significantly suppressed the proliferation and colony formation of EOC cells (Figure S1A–C, Supporting Information while circ_0001546 knockdown enhanced cancer cell viability and clonogenicity (Figure S1D–F, Supporting Information). Wound healing (Figure 2F–H) and Transwell assays (Figure 2I–K) showed that hsa_circ_0001546 overexpression significantly suppressed the migration of EOC cells, and hsa_circ_0001546 knockdown markedly promoted this phenotype. These data suggest that circ_0001546 acts as a suppressor in the progression of EOC.

### 2.3 hsa_circ_0001546 Directly Interacts with 14‐3‐3 Proteins in EOC Cells

**Figure 3:**
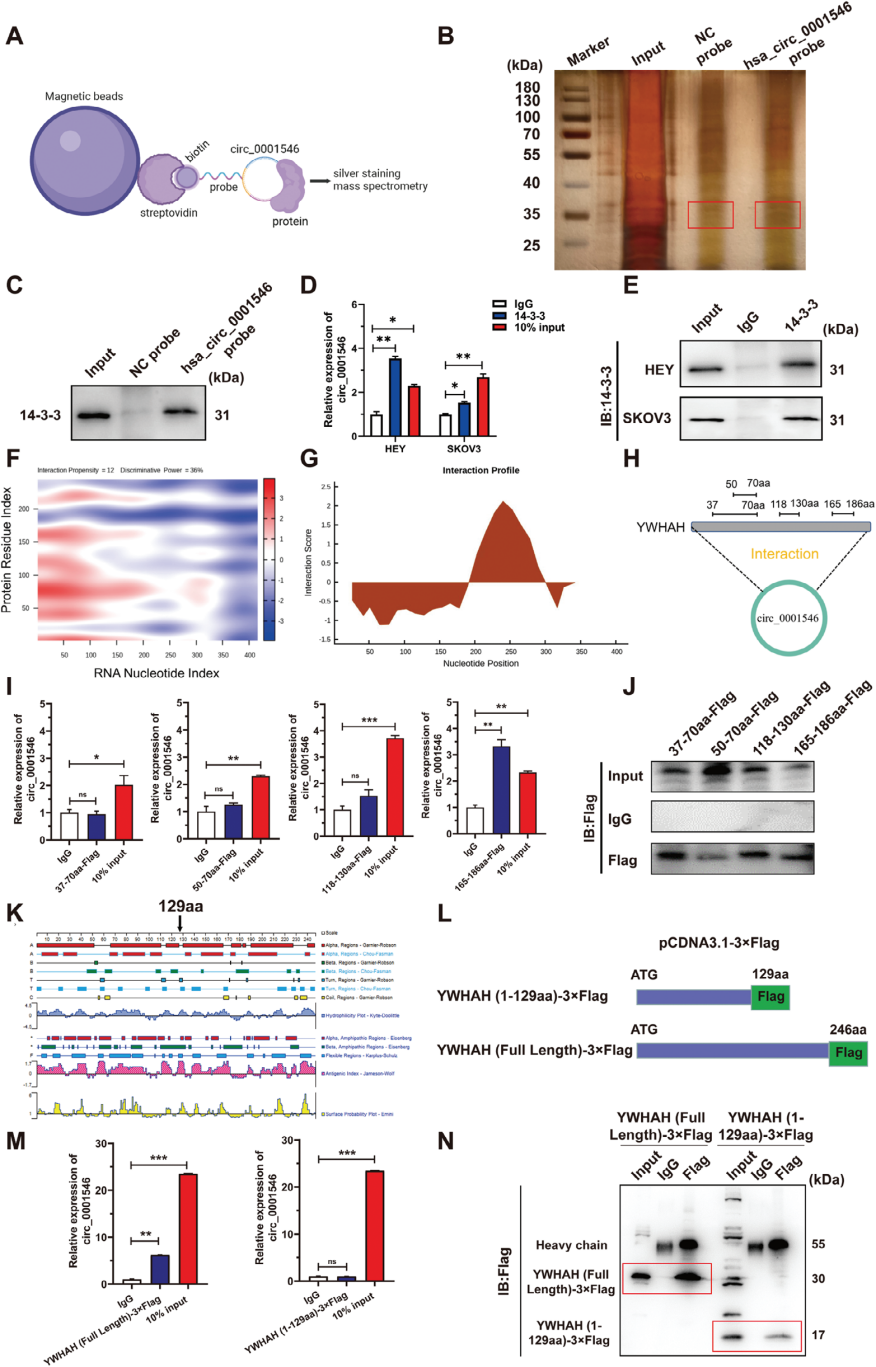
hsa_circ_0001546 directly interacts with 14‐3‐3 proteins to act as their chaperone molecule in EOC cells. A) Schematic illustration of identification potential hsa_circ_0001546‐associated proteins using RNA pull‐down and mass spectrometry analysis. B) Silver staining images of SDS‐PAGE gels, in which hsa_circ_0001546/proteins complexes from RNA pull‐down assays of HEY cells were separated; the red box represents the specific protein bands in pull‐down complexes by hsa_circ_0001546 probe when compared with NC probe. C) RNA pull‐down followed by western blot confirmed the binding of hsa_circ_0001546 and 14‐3‐3 proteins. D) Fold enrichment of hsa_circ_0001546 in HEY and SKOV3 cells was detected by RIP assay using 14‐3‐3 or IgG primary antibody. E) 14‐3‐3 protein immunoprecipitated by 14‐3‐3 antibody or IgG was detected by western blot analysis. F) The specific binding sequence and sites of circ_0001546 and YWHAH was predicted by catRAPID database presented by heatmap analysis (http://www.tartaglialab.com/). G. The interaction profile analysis of binding between different regions of 14‐3‐3 proteins and hsa_circ_0001546 by catRAPID database (http://www.tartaglialab.com/). H) The expressing vectors of different fragments from YWHAH protein (37‐70, 50–70, 118–130, 165–186 aa) were constructed using pCDNA3.1‐Flag vector. I) Fold enrichment of hsa_circ_0001546 in 37–70, 50–70, 118–130, and 165–186 aa regions was detected by RIP assay. J) The binding of Flag proteins to magnetic beads was detected by western blot analysis. K) Protein secondary structure of YWHAH analyzed by DNASTAR software. L) Full‐length and truncated fragments of YWHAH were cloned into pCDNA3.1 vector with 3×Flag tag. M) Fold enrichment of hsa_circ_0001546 in YWHAH (Full length) and YWHAH (1‐129aa) was detected by RIP assay using Flag or IgG primary antibody. N) The binding of Flag proteins to magnetic beads was detected by western blot analysis. ^ns^ *p* < 0.05, ^*^ *p* < 0.05, ^**^ *p* < 0.01, ^***^ *p* < 0.001. Three independent experiments were performed.

To explore the molecular mechanisms underlying the hsa_circ_0001546 regulation of EOC progression, an RNA pull‐down assay was conducted to identify the proteins associated with hsa_circ_0001546 by using the hsa_circ_0001546 probe and NC probe. The precipitates in the RNA pull‐down assay were separated by SDS‒PAGE. After silver staining, the hsa_circ_0001546‐specific band at ≈35 kDa (red box) was excised and analyzed using mass spectrometry (**Figure** **3**A,B; Figure S2A, Supporting Information). We found that hsa_circ_0001546 could bind to 4 members of the 14‐3‐3 protein family, including YWHAB, YWHAH, YWHAQ, and YWHAZ, and the abundance of hsa_circ_0001546 binding to these members was 3–6 times higher than that binding to the NC (Figure S2B, Supporting Information). Therefore, 14‐3‐3 proteins were the candidate proteins binding to hsa_circ_0001546. RNA pull‐down followed by western blotting confirmed the direct binding of hsa_circ_0001546 and 14‐3‐3 proteins (Figure 3C). Furthermore, the RIP assay verified that 14‐3‐3 proteins could enrich hsa_circ_0001546, and the nonspecific result of RT‒qPCR was excluded by western blotting, proving the successful binding of 14‐3‐3 protein antibody to magnetic beads (Figure 3D,E).

Subsequently, we investigated the specific regions of 14‐3‐3 proteins binding to hsa_circ_0001546 by the catRAPID database. The protein sequences of 4 14‐3‐3 protein members were matched with the sequence of hsa_circ_0001546. The prediction results showed that circ_0001546 might bind to the 26–77, 51–102, 76–127, and 151–202 aa regions of 14‐3‐3 proteins, and we chose to display the prediction results of the YWHAH protein because of its highest abundance in mass spectrometry (Figures 3F,G; S2C, Supporting Information). Next, homology analysis was performed on the amino acid sequences of the 4 14‐3‐3 protein members, and these results and the above prediction results suggested that the most likely regions for hsa_circ_0001546 binding in 14‐3‐3 proteins were the 37–70, 50–70, 118–130, and 165–186 aa regions (Figure 3H). Thus, we used the sequence of the YWHAH protein as a template to construct pCDNA3.1‐Flag plasmids, which were named 37–70, 50–70, 118–130, and 165–186 aa‐Flag. The RIP assay revealed that the 165—186 aa region of the YWHAH protein was responsible for the interaction with hsa_circ_0001546, and the nonspecific result of RT‒qPCR was excluded by western blotting (Figure 3I–J). To further verify whether hsa_circ_0001546 binds to 165–186 aa of the YWHAH protein, we analyzed the secondary structure of the YWHAH protein by DNASTAR software to avoid the potential functional domain (Figure 3K). Plasmids with full‐length and truncated sequences with 3×Flag were constructed as YWHAH (Full Length)‐3×Flag and YWHAH (1‐129 aa)‐3×Flag, respectively (Figure 3L). The successful construction of these 2 plasmids, with fragment lengths of 807 bp and 453 bp, respectively, was verified by agarose gel electrophoresis (Figure S2D, Supporting Information). The RIP assay revealed that compared with the IgG group, the enrichment of hsa_circ_0001546 by YWHAH (Full Length)‐3×Flag increased significantly, while the enrichment of hsa_circ_0001546 by YWHAH (1‐129 aa)‐3×Flag showed no significant change (Figure 3M). The results of western blotting showed the successful binding of 14‐3‐3 protein antibody to magnetic beads and excluded the nonspecific result of RT‒qPCR (Figure 3N). These data reveal that hsa_circ_0001546 directly interacts with YWHAH in the 165–186 aa region.

### 2.4 hsa_circ_0001546/14‐3‐3 Proteins Promote Tau Phosphorylation and Induce Tau Aggregation by Recruiting CAMK2D

**Figure 4:**
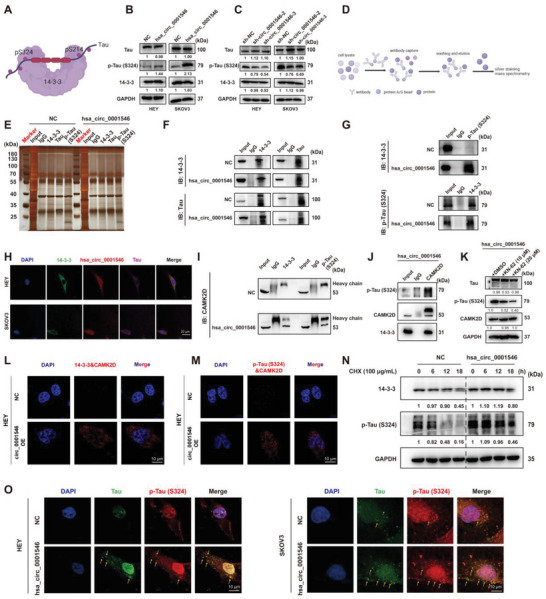
hsa_circ_0001546/14‐3‐3 promotes Tau phosphorylation and induces Tau aggregation via recruiting CAMK2D. A) Schematic diagram of the binding of 14‐3‐3 proteins with Tau proteins to cause its phosphorylation. B) The protein levels of Tau, p‐Tau (S324), and 14‐3‐3 were detected in hsa_circ_0001546 stable‐overexpressing cells. C) The protein levels of Tau, p‐Tau (S324), and 14‐3‐3 were detected in hsa_circ_0001546 stable‐down‐expressing cells. D) Co‐IP assay was performed to verify the combination of 14‐3‐3 and Tau or p‐Tau (S324) in HEY cells transfected with NC and hsa_circ_0001546 overexpressing vector. E) Silver staining image of Co‐IP assays with 14‐3‐3, Tau, p‐Tau (S324), and IgG primary antibodies. F,G) The lysates of HEY cells transfected with NC and hsa_circ_0001546 overexpressing vector were subjected to Co‐IP using 14‐3‐3, Tau, and p‐Tau (S324) primary antibodies as indicated. H) Subcellular co‐localization of hsa_circ_0001546, 14‐3‐3, and Tau detected by IF and FISH assays in HEY and SKOV3 cells. Scale bar = 20 µm. I) Co‐IP assay was performed to verify the combination of 14‐3‐3, p‐Tau (S324) with CAMK2D in HEY cells transfected with NC and hsa_circ_0001546 overexpressing vector. J) The lysates of HEY cells transfected with hsa_circ_0001546 overexpressing vector were subjected to Co‐IP using CAMK2D primary antibody as indicated. K) In hsa_circ_0001546 stable‐overexpressing cells treated with KN‐62, the protein levels of Tau, p‐Tau (S324) and CAMK2D were detected. Representative images are shown. L) PLA assay was performed to verify the direct binding between 14‐3‐3 and CAMK2D in hsa_circ_0001546 stable‐overexpressing and NC cells. Scale bar = 10 µm. M) PLA assay was performed to verify the direct binding between CAMK2D and p‐Tau (S324) in hsa_circ_0001546 stable‐overexpressing and NC cells. Scale bar = 10 µm. N) In hsa_circ_0001546 stable‐overexpressing cells treated with CHX for 0, 6, 12, 18 h, the protein levels of 14‐3‐3 and p‐Tau (S324) were detected. O) Immunofluorescence detection of Tau (Green), p‐Tau (S324, Red), and Tau aggregation (Yellow arrow) in NC/hsa_circ_0001546 stable‐overexpressing HEY and SKOV3 cells. Scale bar = 10 µm. Three independent experiments were performed.

In a previous study, 14‐3‐3 proteins were proven to regulate the Ser324 and Ser214 phosphorylation sites of Tau, which causes the close binding of 14‐3‐3 and Tau proteins.^[^ ^19^ ^]^ Therefore, to study whether hsa_circ_0001546 regulates the phosphorylation level of Tau, we detected the effect of an increase or decrease in hsa_circ_0001546 on the phosphorylation level of the Ser324 and Ser214 sites of Tau (**Figure** **4**A). The results showed that overexpression of hsa_circ_0001546 could significantly promote the expression of p‐Tau (S324) and p‐Tau (S214) in HEY and SKOV3 cell lines, but there was no significant change in the expression of total Tau and 14‐3‐3 proteins (Figures 4B; S3A, Supporting Information). In addition, we verified this finding in the HEY and SKOV3 cell lines with hsa_circ_0001546 knockdown. The results demonstrated that the levels of p‐Tau (S324) and p‐Tau (S214) were significantly decreased, but the knockdown of circ_0001546 did not alter total Tau or 14‐3‐3 protein levels (Figure 4C; Figure S3A, Supporting Information). To explore the mechanism by which hsa_circ_0001546 promotes Tau phosphorylation, we performed a Co‐IP assay using 14‐3‐3, Tau and p‐Tau (S324) primary antibodies separately in the HEY cell line with upregulation of hsa_circ_0001546, followed by silver staining and mass spectrometry analysis (Figure 4D,E; Figure S3B–D, Supporting Information). We verified that 14‐3‐3 could bind with Tau, and there was no difference in this binding between hsa_circ_0001546‐overexpressing cell lines and control cell lines (Figure 4F). However, the binding between 14‐3‐3 and p‐Tau (S324) increased after the overexpression of hsa_circ_0001546, indicating that hsa_circ_0001546 promoted the phosphorylation of Tau bound to 14‐3‐3 (Figure 4G). Additionally, the combination of FISH and IF assays showed that hsa_circ_0001546, 14‐3‐3, and Tau colocalized in the cytoplasm, which further supported the possibility of the interaction of the 3 (Figure 4H). 14‐3‐3 can bind to Tau and other protein kinases at the same time so that Tau can be used as a substrate of various protein kinases, which regulates the phosphorylation of Tau.^[^ ^20^ ^]^ Consequently, we hypothesize that hsa_circ_0001546 promotes Tau phosphorylation by driving the recruitment of protein kinases mediated by 14‐3‐3. Based on the analysis of the mass spectrometric results of the p‐Tau (S324) primary antibody in a Co‐IP assay, we screened the CAMK2D protein, a family member of CAMK II (Figure S3E, Supporting Information). It is suggested that they may be the key phosphokinases by which 14‐3‐3 proteins recruit and phosphorylate Tau downstream of hsa_circ_0001546. We performed a Co‐IP assay using 14‐3‐3 and p‐Tau (S324) primary antibodies separately in the HEY cell line with upregulation of hsa_circ_0001546 and verified that hsa_circ_0001546 can promote the combination of 14‐3‐3 and p‐Tau (S324) with CAMK2D (Figure 4I). Therefore, we performed a Co‐IP assay using a CAMK2D primary antibody in the HEY cell line with upregulation of circ_0001546 and confirmed that hsa_circ_0001546 can promote the combination of 14‐3‐3 and p‐Tau (S324) with CAMK2D (Figure 4J). To further prove the recruitment of CAMK2D induced by hsa_circ_0001546/14‐3‐3, KN‐62, a CAMK II inhibitor that directly binds to the calmodulin binding site of CAMK II, was used to measure the level of Tau phosphorylation. The results showed that the level of p‐Tau (S324) decreased significantly after KN‐62 treatment, while the levels of Tau and CAMK2D did not change significantly (Figure 4K). Besides, the PLA assays were performed to verify that the overexpression of hsa_circ_0001546 could drive the direct binding of 14‐3‐3 and CAMK2D (Figure 4L) as well as CAMK2D and p‐Tau (S324) (Figure 4M). To further explore the mechanism of Tau phosphorylation promoted by hsa_circ_0001546, we carried out a CHX assay. After treatment with 100 µg mL^−1^ CHX for 0, 6, 12, and 18 hours, the stability of 14‐3‐3 and p‐Tau (S324) was detected. The results showed that upregulation of hsa_circ_0001546 could limit the increase in the half‐life of 14‐3‐3, which was accompanied by an increase in the half‐life of p‐Tau (S324) (Figure 4N). Thus, hsa_circ_0001546 promotes the phosphorylation of Tau by improving the stability of 14‐3‐3. Based on the above data, we thought that hsa_circ_0001546 may act as a chaperone molecule of 14‐3‐3 proteins, which are adaptor/scaffold proteins with limited regulation of stability, and further participate in the interaction between other proteins. Abnormal phosphorylation of Tau has been proven to be the critical inducement of Tau aggregation.^[^ ^21^ ^]^ IF assays using Tau and p‐Tau (S324) primary antibodies confirmed that upregulation of hsa_circ_0001546 can increase p‐Tau (S324), which binds to 14‐3‐3 in HEY and SKOV3 cell lines and further promotes the aggregation of Tau, as shown by the yellow arrow (Figure 4O). These data demonstrate that in EOC, hsa_circ_0001546 promotes Tau phosphorylation by driving the recruitment of CAMK2D mediated by 14‐3‐3, which thus changes the phosphorylation state of Tau bound to 14‐3‐3 from nonphosphorylated to phosphorylated (Ser324) and leads to an increase in Tau aggregation that ultimately inhibits the proliferation and metastasis of EOC.

### 2.5 The hsa_circ_0001546/14‐3‐3/CAMK2D/p‐Tau Complex Promotes LPO‐Dependent Ferroptosis in EOC

**Figure 5:**
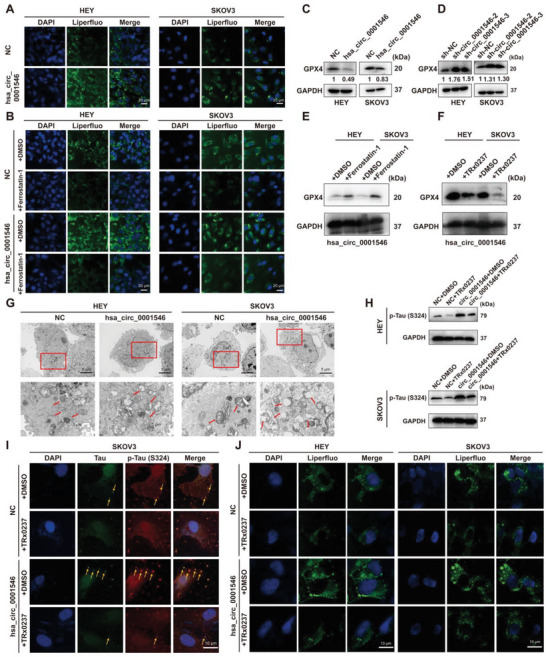
The hsa_circ_0001546/14‐3‐3/CAMK2D/p‐Tau complex promotes LPO‐dependent ferroptosis in EOC. A) Detection of lipid peroxides in NC/hsa_circ_0001546 stable‐overexpressing HEY and SKOV3 cells by Liperfluo. Scale bar = 20 µm. B) Lipid peroxides were detected by Liperfluo in NC/hsa_circ_0001546 stable‐overexpressinsg HEY and SKOV3 cells treated with Ferrostatin‐1. Scale bar = 20 µm. C) The protein level of GPX4 was detected in NC/hsa_circ_0001546 stable‐overexpressing cells. D) The protein level of GPX4 was detected in NC/hsa_circ_0001546 stable‐down‐expressing cells. E) The protein level of GPX4 was detected in hsa_circ_0001546 stable‐overexpressing cells treated by Ferrostatin‐1. F) The protein level of GPX4 was detected in hsa_circ_0001546 stable‐overexpressing cells treated by TRx0237. G) Transmission electron microscopy detection of the morphological changes of mitochondria (red arrow) in hsa_circ_0001546 overexpressing HEY and SKOV3 cells. Representative images are shown. H) In hsa_circ_0001546 stable‐overexpressing HEY and SKOV3 cells treated with TRx0237, the protein level of p‐Tau (S324) was detected. Representative images are shown. I) In hsa_circ_0001546 stable‐overexpressing SKOV3 cells treated with TRx0237, Tau aggregation (yellow arrow) was detected by Immunofluorescence. Scale bar = 10 µm. J) In hsa_circ_0001546 stable‐overexpressing HEY and SKOV3 cells treated with TRx0237, lipid peroxides were detected by Liperfluo. Scale bar = 10 µm. Three independent experiments were performed.

Because Tau is associated with ferroptosis, we further studied whether hsa_circ_0001546 can inhibit the development of EOC by affecting ferroptosis. As the critical cause of ferroptosis, LPOs were first detected by Liperfluo in HEY and SKOV3 cell lines with upregulation of hsa_circ_0001546. After overexpressing hsa_circ_0001546, the number of LPOs in HEY and SKOV3 cell lines was significantly higher than that in control cells (**Figure** **5**A). This means that hsa_circ_0001546 can promote the formation of LPOs in EOC cell lines, which is closely related to ferroptosis. To further verify the above data, the ferroptosis inhibitor ferrostatin‐1 was used to treat cells for 24 hours to detect changes in LPOs. The results showed that the level of LPOs in hsa_circ_0001546‐overexpressing HEY and SKOV3 cells was significantly decreased under ferrostatin‐1 treatment (Figure 5B), indicating that hsa_circ_0001546 could induce ferroptosis by promoting the production of LPOs in EOC cells. Subsequently, we detected the expression of GPX4, one of the marker proteins of ferroptosis, and found that upregulation or downregulation of hsa_circ_0001546 suppressed or boosted the expression of GPX4, which further confirmed the effect of hsa_circ_0001546 on ferroptosis (Figure 5C‐D). Then, in order to explore the mechanism induced by hsa_circ_0001546 in ferroptosis, we treated hsa_circ_0001546 stable‐overexpressing cells using ferrostatin‐1 and TRx0237 respectively. Ferrostatin‐1 is a selective ferroptosis inhibitor to suppress lipid peroxidation. It has also been reported to affect the expression of GPX4.^[^ ^22^ ^]^ TRx0237 (Leucomethylene blue mesylate) is an active second‐generation tau protein aggregation inhibitor and reduces the phosphorylation of Tau.^[^ ^23^ ^]^ However, interestingly, treatment with ferrostatin‐1 could rescue the expression of GPX4 in hsa_circ_0001546‐expressing cells, while treatment with TRx0237 could not, which meant that the ferroptosis induced by the hsa_circ_0001546/14‐3‐3/CAMK2D/p‐Tau complex was LPO‐dependent rather than GPX4‐dependent (Figure 5E,F). Observing the morphological changes of mitochondria in cells is another common method to detect ferroptosis. In the process of ferroptosis, mitochondria usually show atrophy, an increase in bilayer membrane density, breakage of the mitochondrial outer membrane, and a decrease or disappearance of mitochondrial cristae. Therefore, transmission electron microscopy (TEM) was used to observe the morphology of mitochondria in hsa_circ_0001546‐overexpressing HEY and SKOV3 cells, and we found that the mitochondrial outer membrane was intact and that the mitochondrial cristae were clear in the control cells, but rupture of the mitochondrial outer membrane and reduction or even disappearance of mitochondrial cristae could be observed after overexpression of hsa_circ_0001546 (red arrow) (Figure 5G). These results indicate that hsa_circ_0001546 can induce ferroptosis to protect against EOC development. To further explore whether the promoting effect of hsa_circ_0001546 on ferroptosis of EOC cells was affected by Tau phosphorylation, the p‐Tau inhibitor TRx0237 was used to treat hsa_circ_0001546‐overexpressing HEY and SKOV3 cells. The data demonstrated that TRx0237 had a significant inhibitory effect on the protein level of p‐Tau (S324) in hsa_circ_0001546‐overexpressing cell lines (Figure 5H). In addition, when Tau phosphorylation at S324 was inhibited, Tau aggregation decreased significantly, as shown by the yellow arrow (Figure 5I). Subsequently, after treatment with TRx0237, LPOs were detected, and we found that the inhibition of p‐Tau decreased the number of LPOs in hsa_circ_0001546‐expressing cells (Figure 5J). The above data suggested that the hsa_circ_0001546/14‐3‐3/CAMK2D/p‐Tau complex causes Tau aggregation by promoting Tau phosphorylation (S324), which leads to LPO accumulation and finally induces ferroptosis.

### 2.6 hsa_circ_0001546/14‐3‐3 Inhibits EOC Progression In Vivo Via Phosphorylation of Tau

**Figure 6:**
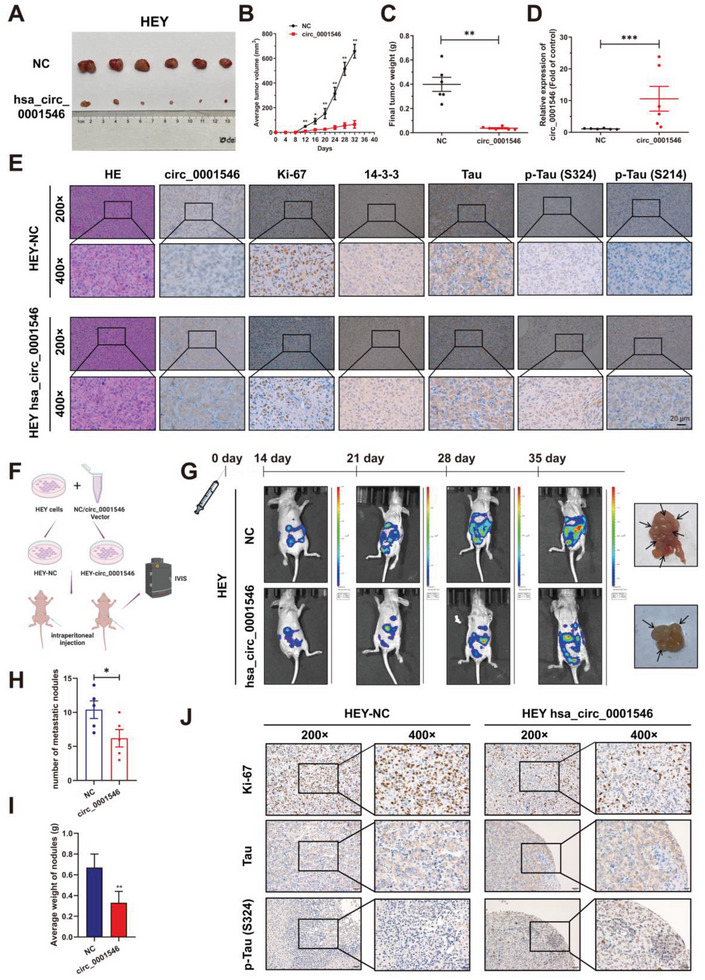
hsa_circ_0001546 suppresses EOC tumor growth and metastasis in vivo. A) Overexpression of hsa_circ_0001546 inhibited cell‐derived xenograft growth in nude mice (6 mice per group). B) Statistical analysis of hsa_circ_0001546 overexpressing tumor volume in nude mice. C) Statistical analysis of hsa_circ_0001546 overexpressing tumor weight in nude mice. D) The expression of hsa_circ_0001546 in hsa_circ_0001546 overexpressing tumor was detected by RT‐qPCR. E) hsa_circ_0001546 overexpressing tumor tissues were stained with hematoxylin and eosin solution. Protein levels of Ki67, 14‐3‐3, Tau, p‐Tau (S324), and p‐Tau (S214) were detected by IHC. RNA level of hsa_circ_0001546 was detected by ISH. Scale bar = 20 µm. F) Graphic illustration of the intraperitoneal injection of NC/hsa_circ_0001546 stable‐overexpressing HEY cells. G) The metastasis and spread of hsa_circ_0001546 overexpressing HEY cells were monitored by IVIS Imaging System. H) The number of metastatic nodules caused by hsa_circ_0001546 overexpressing cells in nude mice was calculated. I) The average weight of metastatic nodules caused by hsa_circ_0001546 overexpressing cells in nude mice was calculated. J) Protein levels of Ki67, Tau, and p‐Tau (S324) were detected by IHC (original magnification × 200 and × 400). Scale bar = 50 and 20 µm. ^*^ *p* < 0.05, ^**^ *p* < 0.01, ^***^ *p* < 0.001. Three independent experiments were performed.

To investigate the antitumor role of hsa_circ_0001546, we generated xenograft mouse models by subcutaneous injection of HEY cells (5 × 10^6^) expressing hsa_circ_0001546 or NC into nude mice and measured tumor volumes every 4 days (**Figure** **6**A). The results showed that upregulation of hsa_circ_0001546 significantly suppressed tumor growth in mice within 32 days (Figure 6B,C). Moreover, the levels of hsa_circ_0001546 in xenograft tumors were tested by RT‒qPCR (Figure 6D). Additionally, using ISH and IHC, we showed a decrease in Ki67, increases in p‐Tau (S324), p‐Tau (S214), and hsa_circ_0001546, and no changes in 14‐3‐3 and Tau expression in hsa_circ_0001546‐overexpressing tumor tissues (Figure 6E). To study the effect of hsa_circ_0001546 on EOC metastasis, we established an abdominal metastasis model by intraperitoneal injection of HEY cells (5×10^6^) expressing hsa_circ_0001546 or NC into nude mice (Figure 6F). IVIS imaging was used to assess the spread of HEY cells, and the fluorescent signals showed that hsa_circ_0001546 could inhibit the intraperitoneal implantation metastasis of EOC and reduce the number and weight of metastatic nodules (Figure 6G–I). Additionally, using IHC, we showed that Ki67 levels were decreased and p‐Tau (S324) levels were increased in hsa_circ_0001546‐overexpressing tumor tissues, while there was no significant change in Tau expression (Figure 6J).

### 2.7 The hsa_circ_0001546/14‐3‐3/CAMK2D/p‐Tau Complex Inhibits EOC Spread In Vivo by Inducing LPO‐Dependent Ferroptosis

**Figure 7:**
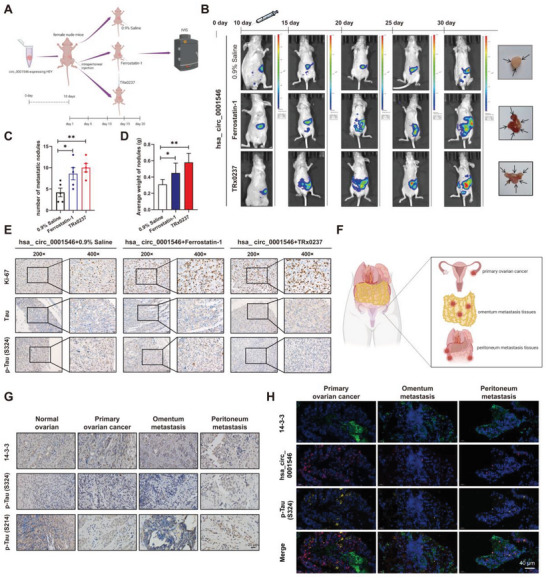
The hsa_circ_0001546/14‐3‐3/CAMK2D/p‐Tau complex inhibits EOC tumor spread in vivo via inducing Tau‐related ferroptosis. A) Graphic illustration of the intraperitoneal injection of 0.9% Saline and Ferrostatin‐1 or intragastric administration of TRx0237 in abdominal metastatic tumor model. B) The number of metastatic nodules caused by hsa_circ_0001546 overexpressing cells treated with Ferrostatin‐1 or TRx0237 in nude mice was calculated. C) The average weight of metastatic nodules caused by hsa_circ_0001546 overexpressing cells treated with Ferrostatin‐1 or TRx0237 in nude mice was calculated. D) Tumor images monitored by IVIS Imaging System of three‐group mice 30 days after intraperitoneal injection and intragastric administration, including 0.9% Saline, Ferrostatin‐1, TRx0237 (5 mice per group). E) Protein levels of Ki67, Tau, and p‐Tau (S324) were detected by IHC (original magnification × 200 and × 400). Scale bar = 50 and 20 µm. F) Graphic illustration of the primary ovarian cancer, omentum metastasis tissues, and peritoneum metastasis tissues. G) Protein levels of 14‐3‐3, p‐Tau (S324), and p‐Tau (S214) were detected by IHC in normal ovarian tissues, primary ovarian cancer, omentum metastasis tissues, and peritoneum metastasis tissues. Scale bar = 20 µm. H) Subcellular co‐localization and expression of hsa_circ_0001546 (Red), 14‐3‐3 (Green), and p‐Tau (S324, Yellow) detected by IF and FISH assays in normal ovarian tissues and paired primary ovarian cancer, omentum metastasis, and peritoneum metastasis tissues. Scale bar = 40 µm.

To investigate whether hsa_circ_0001546 inhibits EOC metastasis by inducing ferroptosis, we treated hsa_circ_0001546‐overexpressing cells with ferrostatin‐1 and TRx0237 respectively, and the results showed that ferrostatin‐1 and TRx0237 could rescue the inhibitory effect of hsa_circ_0001546 on cell migration and invasion (Figure S4, Supporting Information). Subsequently, we first established an abdominal metastasis model by intraperitoneal injection of HEY cells (5×10^6^) expressing hsa_circ_0001546 into nude mice. On the 10^th^ day after intraperitoneal injection, the tumorigenesis of HEY cells was observed by IVIS. After successful establishment, we randomly divided the mice into 3 groups: 0.9% saline, ferrostatin‐1, and TRx0237 (**Figure** **7**A). Mice were injected or orally administered treatments every 5 days, and the spread or metastasis of HEY cells was detected by IVIS imaging. The data showed that both ferrostatin‐1 and TRx0237 blocked the inhibitory effect of hsa_circ_0001546 on the spread or metastasis of EOC and caused more obvious intraperitoneal implantation metastasis compared with the 0.9% saline group (Figure 7B). In addition, treatment with ferrostatin‐1 and TRx0237 increased the number and weight of metastatic nodules in the abdominal cavity of mice (Figure 7C,D). The IHC results supported that compared with the 0.9% saline group, the other 2 groups had higher levels of Ki‐67 and lower levels of p‐Tau (S324), and Tau expression was not significantly changed (Figure 7E). These results indicated that the hsa_circ_0001546/14‐3‐3/CAMK2D/p‐Tau complex inhibits EOC metastasis by inducing ferroptosis.

To further clarify the function of hsa_circ_0001546, 14‐3‐3, and p‐Tau in EOC, different EOC tissue species from patients were used to verify the previous results (Figure 7F). The IHC results showed that the expression of 14‐3‐3 was not obviously different between normal ovarian tissues and paired primary ovarian cancer, omentum metastasis and peritoneum metastasis tissues, while the expression of p‐Tau (S324) and p‐Tau (S214) was decreased significantly in paired primary ovarian cancer, omentum metastasis and peritoneum metastasis tissues compared with normal ovarian tissues and was lowest in omentum metastasis and peritoneum metastasis tissues (Figure 7G). In addition, the combination of FISH and IF assays showed that the levels of p‐Tau (S324), p‐Tau (S214), and hsa_circ_0001546 were gradually decreased in primary ovarian cancer, omentum metastasis, and peritoneum metastasis tissues, and the levels of these factors were positively correlated (Figure 7H; Figure S5, Supporting Information).

## 3 Discussion

EOC metastasis often occurs in adjacent organs, and this spread in the peritoneal cavity is linked with ascites formation and OC cell shedding.^[^ ^24^ ^]^ In addition, considering possible pelvic and paraaortic lymph node involvement, distant metastasis also occurs in advanced EOC.^[^ ^25^ ^]^ For EOC metastasis, traditional debulking surgery in combination with platinum‐taxane chemotherapy sometimes fails to have an ideal prognosis, and patients can benefit from targeted therapy drugs, such as poly‐(ADP‐ribose) polymerase (PARP) inhibitors.^[^ ^26^ ^]^ In current clinical treatment, more emphasis has been placed on individual genetic diversity, which means that the same drug may have opposite effects on different patients.

Benefiting from their specific structure, circRNAs show more obvious stability than other linear ncRNAs, which means that they are persistently regulated at the cellular level. Recent evidence has proven that circRNAs are involved in various regulatory mechanisms, such as splicing of linear mRNA counterparts, regulating transcription of parental genes, acting as miRNA sponges, and interacting with associated proteins and peptides.^[^ ^27^ ^]^ In this paper, we verified that hsa_circ_0001546 is spliced from FAM114A2 exons 2–4 and downregulated in EOC primary and metastatic tissues. Interestingly, we found that hsa_circ_0001546 acts as the chaperone molecule of 14‐3‐3, which is an adaptor/scaffold protein participating in the interaction between other proteins. Based on our data, we propose that the indirect scaffold function of circRNAs is sometimes performed by circRNAs interacting with scaffold proteins, rather than by circRNAs alone. This conclusion is much different from other studies illustrating the direct scaffold function of circRNAs in circRNPs.^[^ ^28^ ^]^

The 14‐3‐3 protein has been verified to have 7 different isoforms, which are highly conserved encoding genes in humans.^[^ ^12^ ^]^ In this study, since it had the highest abundance, YWHAH (14‐3‐3 η) was chosen as the representative research object, and the results showed that hsa_circ_0001546 directly bound to the 165–181 aa region of YWHAH, whose sequence was MQPTHPIRLGLALNFSVFYYEI. However, this fragment also existed on the 162–183 aa of YWHAB (14‐3‐3 β) and 160–181 aa of YWHAQ (14‐3‐3 θ) and YWHAZ (14‐3‐3 ζ), which means that hsa_circ_0001546 can bind to several members of the 14‐3‐3 protein family. Since the main natural forms of 14‐3‐3 protein are homodimers and heterodimers, they can interact with other client proteins and cause conformational changes.^[^ ^11^ ^]^ This binding is dependent on the phosphorylation of the central serine and induces the conditional association between 14‐3‐3 and protein ligands containing motif RSXpSXP.^[^ ^29^ ^]^ Based on this mechanism, 14‐3‐3s can recruit a series of phosphokinases, including protein kinase C (PKC),^[^ ^30^ ^]^ glycogen synthase kinase‐3β (GSK3β)^[^ ^31^ ^]^ and leucine‐rich repeat protein kinase 2 (LRRK2).^[^ ^32^ ^]^ We confirmed that the expression of hsa_circ_0001546 can drive the recruitment of CAMK2D mediated by 14‐3‐3s.

The close interaction between 14‐3‐3 and Tau proteins has been proven to play key roles in AD^[^ ^20^ ^]^ and PD.^[^ ^33^ ^]^ 14‐3‐3 can bind to Tau and protein kinase simultaneously, which makes Tau a more suitable substrate for various kinases.^[^ ^19^ ^]^ Our study found that hsa_circ_0001546 promoted the direct binding between 14‐3‐3, CAMK2D, and Tau and then caused abnormal phosphorylation of the Tau protein at the Ser324 site. Following this regulation, the status of Tau protein bound to 14‐3‐3 proteins changes from nonphosphorylated to phosphorylated and finally induces further Tau aggregation. Abnormal phosphorylation and mutation are the major contributing factors to increased Tau aggregation.^[^ ^34^ ^]^ Tau aggregation is a dynamic complex composed of various RNAs, DNAs, and proteins and is thought to be one of the major inducers of a series of neurodegenerative disorders.^[^ ^35^ ^]^ Continuous Tau aggregation can form neurofibrillary tangles (NFTs) and cause aberrant cell death in neurons,^[^ ^36^ ^]^ and a similar regulatory mechanism was observed in EOC cells in our study. Based on our results, the 14‐3‐3/CAMK2D/Tau complex driven by hsa_circ_0001546 caused Tau aggregation‐dependent LPO accumulation and ferroptosis. We verified the relationship between Tau aggregation and ferroptosis in EOC cells, which also exist in neurons,^[^ ^37^ ^]^ and cell death was ultimately observed in both cell types. However, based on our conclusion, the ferroptosis caused by Tau aggregation has opposite effects on EOC cells and neurons, and it means that the overexpression of hsa_circ_0001546 for EOC therapeutics may cause potential side effects on other organs, which deserves further study. In addition, interestingly, although the ferroptosis marker glutathione peroxidase 4 (GPX4) was downregulated in hsa_circ_0001546‐expressing EOC cells, the rescue assay showed that the level of GPX4 had no correlation with the 14‐3‐3/CAMK2D/Tau complex, which means that there is another molecular mechanism involving hsa_circ_0001546 and GPX4‐dependent ferroptosis in EOC.

Based on our data, we also established an EOC xenograft model by intraperitoneal injection of hsa_circ_0001546‐overexpressing cells and treated them by intragastric administration of the Tau inhibitor TRx0237 and intraperitoneal administration of the ferroptosis inhibitor ferrostatin‐1. The results showed that both can block the suppressive effect of hsa_circ_0001546 on EOC progression and cause more obvious peritoneal dissemination. Although Tau showed a negative effect in AD, PD, and breast cancer,^[^ ^38^ ^]^ its opposite function can be observed in IDH1‐mutant glioma^[^ ^7^ ^]^ and hsa_circ_0001546‐downregulated EOC, which means that genetic differences in various diseases should be considered in the clinical use of Tau inhibitors. In addition, the occurrence of ferroptosis driven by Tau was simultaneously verified in neurons and EOC cells, and programmed cell death was observed in both cell types.

**Figure 8:**
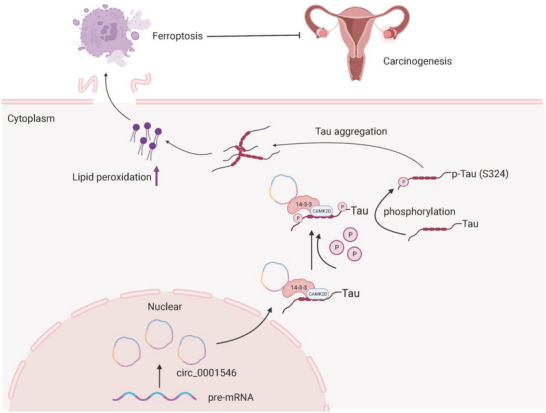
Graphic illustration of the hsa_circ_0001546/14‐3‐3/CAMK2D/p‐Tau complex inhibiting EOC spread via driving Tau aggregation‐dependent LPOs accumulation and ferroptosis.

In summary, our data provide new evidence for the existence of the hsa_circ_0001546/14‐3‐3/CAMK2D/Tau complex, which causes the abnormal phosphorylation and aggregation of Tau and ultimately drives Tau aggregation‐dependent LPO accumulation and ferroptosis (**Figure** **8**). Based on our results, the use of a Tau inhibitor in hsa_circ_0001546‐downregulated EOC can promote EOC progression. Therefore, in our opinion, the hsa_circ_0001546/14‐3‐3/CAMK2D/Tau complex plays an important role in Tau aggregation‐dependent ferroptosis, and its occurrence should be considered in the clinical application of Tau inhibitors. Considering the opposite effects of Tau aggregation‐dependent ferroptosis in EOC cells and neurons, the different effects in various organs caused by the clinical usage of Tau inhibitors should be paid more attention. In clinical treatment, making full use of existing drugs in cancers with different molecular subtypes may be a more realistic strategy than developing novel drug targets.

## 4 Experimental Section

### EOC Clinical Specimens

Eight normal ovarian tissues and 10 EOC tissues were obtained from the Yunnan Cancer Hospital, Department of Gynecology (Yunnan, China), and were approved by the ethics committee of Yunnan Cancer Hospital. Sixty‐nine normal ovarian tissues and 68 EOC tissues were collected for RNA extraction and RT‒qPCR analysis. These tissue samples were obtained from Fudan University Shanghai Cancer Center, and ethical approval was granted by the ethics committee of Fudan University Shanghai Cancer Center. The normal ovarian tissues came from patients with other kinds of cancers and total hysterectomy. Cases from the Kaplan‒Meier Plotter Database were collected to analyze overall and progression‐free survival rates. The detailed clinical characteristics are shown in Table S1 (Supporting Information).

### Cell Lines and Cell Culture

The human normal ovarian epithelial cell line HOSEpiC; human ovarian epithelial cancer cell lines OVCAR8, TOV‐112D, A2780, HO8910, HEY, and SKOV3; and lentiviral packaging cells HEK293T were obtained from American Type Culture Collection (ATCC, USA) or the Cell Bank, China Academy of Sciences (Shanghai, China). The SKOV3 cell line was cultured in RPMI‐1640 medium (Corning Cellgro, Manassas, VA, USA), and the other cell lines were cultured in Dulbecco's modified Eagle's medium (Corning Cellgro, Manassas, VA, USA). All media were supplemented with 10% FBS (Gibco, Gaithersburg, MD, USA) and an antibiotic cocktail of 100 U mL^−1^ penicillin and 100 µg mL^−1^ streptomycin (HyClone, Logan, UT, USA). Cells were cultured at 37 °C with 5% CO_2_.

### RNA Extraction and Reverse Transcription‐Quantitative Real‐Time PCR (RT‒qPCR) analysis

Total RNA of tissues and cell samples was extracted by TRIzol UP (TransGen Biotech, Beijing, China) following the manufacturer's instructions. The PrimeScript RT reagent Kit with gDNA Eraser Kit (TaKaRa Biotech, Dalian, China) was used to construct a cDNA library of circRNAs. The primers were designed to cover the junction sites for hsa_circ_0001546, and the circRNA level was quantified by RT‒qPCR using Hieff qPCR SYBR Green Master Mix (Yeasen Biotech, Shanghai, China) with 18S RNA as the endogenous control. The relative quantification (2^−ΔΔCT^) method was used to analyze the data. Information on the primers is listed in Table S2 (Supporting Information).

### RNase R Treatment

RNA samples were incubated at 37 °C for 90 min with 6 U/µg RNase R. After reverse transcription, 1% agarose gel electrophoresis was used to detect the expression and molecular weight of each target gene after PCR with divergent primers and convergent primers. Information on the primers is listed in Table S2 (Supporting Information).

### Cell Transfection

For gene overexpression and knockdown experiments, cells were seeded on 6 cm plates and transfected with oligonucleotides using Lipofectamine 2000 (Thermo Fisher Scientific, Boston, MA, USA) according to the manufacturer's instructions. For stable expression, the lentiviral vector containing the desired gene, together with the packing plasmid pSPAX2 and envelope plasmid pMD2.G, were transfected into HEK293T cells at a ratio of 4:3:1. The cell supernatants containing lentiviral particles were collected and used to infect EOC cell lines with polybrene (8 µg mL^−1^). Puromycin (2 µg mL^−1^) was added to select the positive cells.

### Cell Proliferation Assay

For the CCK‐8 assay, EOC cells were seeded into a 96‐well plate (Corning, Incorporated, New York, USA) at a density of 1.5×10^3^ cells per well. Five percent CCK‐8 (Yeasen Biotech, Shanghai, China) was added to each well and incubated for 2 h at 37 °C in a 5% CO_2_ environment. Finally, the absorbance of different groups was measured at a wavelength of 450 nm at 24, 48, 72, and 96 h.

For the colony formation assay, 600 cells were plated in each well of a 6‐well plate (Corning, Incorporated, New York, USA) and continuously cultured for 12–14 days at 37 °C with 5% CO_2_. Colonies were first fixed using methanol at room temperature for 15 min, stained with crystal violet (0.5% w/v) at room temperature for 30 min, and then counted.

### Wound Healing Assay

EOC cells were plated into a 12‐well plate (Corning, Incorporated, New York, USA) until the density reached 100%. After treatment with Mitomycin C (10 µg mL^−1^) for 2 h, a single‐scratch wound was introduced through the middle of each well with a sterile pipette tip. Next, cells were incubated with FBS‐free medium for 24 or 36 h, and the migration was assessed and photographed by microscopy.

### Transwell Assay

A total of 1 × 10^4^ EOC cells were seeded into a Boyden chamber insert (BD Biosciences, New Jersey, USA) in a 24‐well culture plate (Corning, Incorporated, New York, USA) and cultured in 200 µL FBS‐free medium. Medium containing 30%–50% FBS was added to the bottom chamber. After incubation for 24–48 h, the invaded cells were fixed in methanol for 20–30 min, stained with crystal violet (0.5% w/v) for 30 min, and counted under a microscope. For invasion assay, Matrigel Matrix (356234, Corning, Incorporated, New York, USA) was spread on the upper layer, and the remaining steps were performed as described above.

### Western Blotting

Total protein was obtained from EOC cells using RIPA lysis buffer (Leagene Biotechnology, Beijing, China) and quantified using a Protein BCA Assay Kit (Beyotime, Shanghai, China). The protein was transferred to a polyvinylidene difluoride (PVDF) membrane (Millipore Corporation, Billerica, MA, USA) following sodium dodecyl sulfate‒polyacrylamide gel electrophoresis and then blocked in 5% nonfat powdered milk for 1.5 h at room temperature followed by incubation at 4 °C overnight with anti‐14‐3‐3, anti‐Tau, anti‐Flag (DYKDDDDK), anti‐GPX4, anti‐CAMK2D (14503‐1‐AP/66499‐1‐Ig/20543‐1‐AP/67763‐1‐Ig/20667‐1‐AP, 1:1000, Proteintech, Wuhan, China), anti‐phospho‐Tau (Ser324) (ab109401, 1:1000, Abcam, Burlingame, USA), anti‐phospho‐Tau (Ser214) (#11109, 1:1000, Signalway Antibody, Californian, USA), anti‐GAPDH (AC001, 1:1000, ABclonal, Wuhan, China), and anti‐Lamin B1 (13435, 1:1000, Cell Signaling Technology, Boston, MA, USA) antibodies. After 14 h of incubation, the PVDF membrane was washed with Tris‐buffered saline Tween 20 (TBST) 4 times and incubated with a goat anti‐rabbit (AS014, 1:2000, ABclonal, Wuhan, China) or goat anti‐mouse (AS003, 1:2000, ABclonal, Wuhan, China) secondary antibody conjugated to horseradish peroxidase (HRP) for 1.5 h at room temperature before washing with TBST 3 times. Protein bands were visualized with a chemiluminescent HRP substrate (Millipore Corporation, Billerica, MA, USA) and exposed using an E‐Gel Imager (Tanon, Shanghai, China).

### Fluorescence In Situ Hybridization (FISH) Assay

Specific FISH probes targeting hsa_circ_0001546 were designed by GenePharma (Shanghai, China), and the assay was performed using the Fluorescent In Situ Hybridization Kit (GenePharma, Shanghai, China) following the manufacturer's instructions. All stained cells were examined and photographed using a Zeiss LSM880 confocal fluorescence microscope (Carl Zeiss, Jena, Germany). The probe used for FISH is described in Table S2 (Supporting Information).

### Cellular Fractionation

HEY and SKOV3 cells were prepared for the isolation of subcellular fractions to detect hsa_circ_0001546 expression in the cytoplasm and nucleus, and the assay was performed using the Ambion PARIS Kit (AM1921, Thermo Fisher Scientific, Boston, MA, USA) following the manufacturer′s instructions. RNA was extracted from the cytoplasmic and nuclear fractions and subjected to RT‐qPCR. 18S rRNA was used as a cytoplasmic marker, and U6 snRNA was used as a nuclear marker. Protein extraction was simultaneously conducted to verify the purification quality and detected by WB. GAPDH was used as a cytoplasmic marker protein, and Lamin B1 was used as a nuclear marker protein.

### Immunofluorescence (IF) Assay

EOC cells were washed 3 times with PBS and fixed in methanol for 15 min. Fixed cells were washed 3 times with PBS and treated with 0.5% Triton X‐100 for 10 min at 4 °C. After treatment, the cells were washed 3 times with PBS before being blocked in 5% BSA at 4°C overnight. For double staining, the samples were incubated in a mixture of 2 primary antibodies, including 14‐3‐3 (14503‐1‐AP, 1:100, Proteintech, Wuhan, China), Tau (sc‐32274, 1:50, Santa Cruz Biotechnology, CA, USA), and anti‐phospho‐Tau (Ser324) (ab109401, 1:50, Abcam, Burlingame, USA) antibodies, at 4 °C overnight. After incubation, the cells were washed 3 times with phosphate‐buffered saline Tween 20 (PBST) and incubated in a mixture of 2 secondary antibodies at room temperature for 1.5 h before washing with PBST 3 times repeatedly and finally mounting with DAPI Fluoromount‐G (SouthernBiotech, Birmingham, AL, USA). The secondary antibodies included Alexa Fluor 488 AffiniPure goat anti‐rabbit IgG (111‐545‐003, 1:200, Jackson ImmunoResearch, West Grove, PA, USA), Alexa Fluor 488 AffiniPure goat anti‐mouse IgG (115‐545‐003, 1:200, Jackson ImmunoResearch, West Grove, PA, USA), Alexa Fluor 594 AffiniPure goat anti‐rabbit IgG (111‐585‐003, 1:200, Jackson ImmunoResearch, West Grove, PA, USA), and Alexa Fluor 647 AffiniPure goat anti‐mouse IgG (115‐645‐003, 1:200, Jackson ImmunoResearch, West Grove, PA, USA). All stained cells were examined and photographed using a Zeiss LSM880 confocal fluorescence microscope (Carl Zeiss, Jena, Germany).

### Actinomycin D Assays

EOC cells were seeded in a 6‐well plate (Corning, Incorporated, New York, USA) at a density of 70%–80%. Twenty‐four hours later, the cells were exposed to 2 µg mL^−1^ actinomycin D (MedChemExpress, Monmouth Junction, NJ, USA) and collected at the indicated time points. RNA stability was analyzed using RT‒qPCR and normalized to the values measured in the mock treatment group (the 0 h group).

### RNA Pull‐Down and Mass Spectra Analysis

The interaction between hsa_circ_0001546 and proteins was detected with the Pierce Magnetic RNA‒Protein Pull‐Down Kit (20164Y, Thermo Fisher Scientific, Boston, MA, USA) according to the manufacturer's protocols. Biotin‐labeled probes targeting the junction site of hsa_circ_0001546 were synthesized by GenePharma (Shanghai, China), and an oligo probe was used as a control. The cells were lysed by RIPA lysis buffer (Leagene Biotechnology, Beijing, China) and incubated with a biotin‐labeled hsa_circ_0001546 probe after combining with streptavidin magnetic beads. Finally, interacting proteins were identified by silver staining using the Fast Silver Stain Kit (P0017S, Beyotime, Shanghai, China), and mass spectrometry was performed by Applied Protein Technology (Shanghai, China).

### RNA Binding Protein Immunoprecipitation (RIP) Assay

EOC cells were scraped, collected, and stored in lysis buffer at −80 °C overnight, and the RIP assay was performed using a Magna RIP RNA‐Binding Protein Immunoprecipitation Kit (17‐701, Merck KGaA, Darmstadt, Germany), 14‐3‐3 primary antibody (Proteintech, Chicago, USA) and Flag (DYKDDDDK) primary antibody (Proteintech, Chicago, USA) following the manufacturer's instructions. The cDNA library of circRNAs was constructed using the PrimeScript RT reagent Kit with gDNA Eraser (TaKaRa Biotech, Dalian, China). The RNA level of hsa_circ_0001546 was measured by RT‒qPCR, and the protein levels of 14‐3‐3 and Flag were detected by Western blotting.

### Co‐Immunoprecipitation (Co‐IP) Assay and Mass Spectra Analysis

EOC cells were scraped and treated with IP lysis buffer (Leagene Biotechnology, Beijing, China), and the CoIP assay was performed using Protein A/G Immunoprecipitation MagBeads (B23202, Bimake, Shanghai, China). and normal rabbit IgG (PP64B, 2 µg, Merck KGaA, Darmstadt, Germany), 14‐3‐3 (14503‐1‐AP, 2 µg, Proteintech, Wuhan, China), Tau (ab254256, 2 µg, Abcam, Burlingame, USA), and phospho‐Tau (Ser324) (ab109401, 2 µg, Abcam, Burlingame, USA) primary antibodies following the manufacturer's instructions. The protein was detected by sodium dodecyl sulfate‒polyacrylamide gel electrophoresis and treated with a Fast Silver Stain Kit (P0017S, Beyotime, Shanghai, China). The specific bands were cut and sent to Shanghai Biotree Biomedical Technology (Shanghai, China) for mass spectral analysis.

### Lipid Peroxide Measurement

To detect lipid peroxides, EOC cells were exposed to Liperfluo (Dojindo, Kumamoto, Japan) at a final concentration of 20 µM for 1 h at 37 °C with 5% CO_2_ in accordance with the manufacturer's instructions. The cells were examined and photographed using a Zeiss LSM880 confocal fluorescence microscope (Carl Zeiss, Jena, Germany).

### Transmission Electron Microscopy

Treated cells were fixed for 5 min with 2.5% glutaraldehyde solution and collected using cell scrapers. After centrifugation, the cells were pelleted and fixed with 2.5% glutaraldehyde solution for 30 min at room temperature in the dark. Fixed cells were washed with 0.1m sodium phosphate buffer (pH 7.2) and fixed in osmium tetroxide followed by dehydration and embedding. Thin sections were cut, and after staining with uranyl acetate and lead citrate, the sections were examined with a HITACHI HT 7800 transmission electron microscope (TEM) system.

### Cycloheximide Chase Assay

EOC cells were seeded in a 6‐well plate (Corning, Incorporated, New York, USA) at a density of 80%–90%. After adding cycloheximide (CHX) at a final concentration of 100 µg mL^−1^ for 0, 6, 12, and 18 h, the protein was collected and analyzed by Western blotting.

### In Situ Proximity Ligation Assay (PLA)

The in‐situ PLA assay was performed using a Duolink In Situ Red PLA Kit (DUO92101, Sigma‐Aldrich, Saint Louis, MO, USA) following the manufacturer's instructions. The used antibodies were anti‐14‐3‐3 (14503‐1‐AP, 1:100, Proteintech, Wuhan, China), anti‐CAMK2D (sc‐100362, 1:50, Santa Cruz Biotechnology, CA, USA), and anti‐phospho‐Tau (Ser324) (ab109401, 1:50, Abcam, Burlingame, USA). The PLA signal was visualized using a OLYMPUS FV3000 confocal fluorescence microscope (Olympus, Japan).

### Subcutaneous Tumor Xenograft Assay

Five‐week‐old female nude mice were obtained from Gempharmatech Co., Ltd. (Nanjing, China) and fed in specific‐pathogen‐free conditions for further experiments. Animals were randomly assigned to 2 groups. A total of 5 × 10^6^ HEY cells stably transfected with NC/hsa_circ_0001546 (resuspended in 100 µL PBS) were inoculated subcutaneously into the right flank of each mouse. Tumor size was measured with calipers every 4 days, and the following formula was used to calculate tumor volumes: volume = length×width^2^/2. Mice were sacrificed 4 weeks post‐injection and the xenograft tumors were excised and weighed. The experimental protocols were approved by the rules of the Science and Technology Ethics Committee of Shanghai University.

### Intraperitoneal Tumor Xenograft Assay

Five‐week‐old female nude mice were purchased from Gempharmatech Co., Ltd. (Nanjing, China) and fed in specific‐pathogen‐free conditions for further experiments. Animals were randomly assigned to 2 groups. A total of 5 × 10^6^ HEY cells stably transfected with NC/hsa_circ_0001546 (resuspended in 100 µL PBS) were injected into the abdominal cavity of each mouse. Two weeks after the injection, the mice were imaged using an IVIS spectrum imaging system 15 min after intraperitoneal injection of d‐luciferin potassium salt (Yeasen Biotech, Shanghai, China) to monitor abdominal cavity metastasis every 7 days. The mice were humanely euthanized after 35 days, and the abdominal metastatic tumors were enucleated. In addition, 10 days after transplantation of 5 × 10^6^ HEY cells stably transfected with hsa_circ_0001546, mice were randomized into 3 groups (*n* = 5 for each group) for treatment initiation: 1) 0.9% saline (100 µL per mouse, intraperitoneal injection, every 5 days); 2) ferrostatin‐1 (MedChemExpress, Monmouth Junction, NJ, USA, 0.1 mg per mouse, intraperitoneal injection, every 5 days); and 3) TRx0237 (MedChemExpress, Monmouth Junction, NJ, USA, 0.5 mg per mouse, intragastric administration, every 5 days). Ten days after the injection, the mice were imaged using an IVIS spectrum imaging system 15 min after intraperitoneal injection of d‐luciferin potassium salt (Yeasen Biotech, Shanghai, China) to monitor abdominal cavity metastasis every 5 days. The mice were humanely euthanized after 30 days, and the abdominal metastatic tumors were enucleated.

### Immunohistochemistry

After fixation in 10% formalin, primary tumor tissues were embedded in paraffin. Samples were cut into sections of 4 µm thickness. The sections were deparaffinized and dehydrated through xylene and graded alcohols and then rehydrated with demineralized water. After being placed in a boiled antigen retrieval buffer for 5 min, tissue sections were incubated in 3% H_2_O_2_ solution for 25 min at room temperature in the dark. Next, the sections were treated with primary antibodies against Ki67 (GB111141, 1:200, Servicebio, Wuhan, China), 14‐3‐3 (14503‐1‐AP, 1:40, Proteintech, Wuhan, China), Tau (66499‐1‐Ig, 1:400, Proteintech, Wuhan, China), phospho‐Tau (Ser324) (ab109401, 1:40, Abcam, Burlingame, USA), and phospho‐Tau (Ser214) (#11109, 1:50, Signalway Antibody, Californian, USA), followed by the application of goat anti‐rabbit horseradish peroxidase (HRP)‐conjugated antibodies, staining with a 3,3′‐diaminobenzidine reaction solution, and imaging using a digitalized microscope camera.

### In Situ Hybridization (ISH) Assay

After fixation in 10% formalin, primary tumor tissues were embedded in paraffin. Samples were cut into sections of 4 µm thickness. The sections were deparaffinized and dehydrated through xylene and graded alcohols and then rehydrated with RNase‐free water. After being placed in Proteinase K at 37 °C for 20 min, tissue sections were incubated in 3% H_2_O_2_ solution for 15 min at room temperature in the dark. Next, the sections were incubated with prehybridization solution at 37°C for 1 h and treated with hsa_circ_0001546 ISH probe solution at 37 °C overnight, followed by the application of anti‐DIG‐HRP‐conjugated antibodies, staining with a 3,3′‐diaminobenzidine reaction solution, and imaging using a digitalized microscope camera.

### Statistical Analysis

All the experiments in this study were repeated 3 times or more independently to ensure the accuracy of the experimental results. GraphPad Prism 8 was used to draw charts, and ImageJ was used to analyze the experimental data. The mean ± standard deviation (SD) was used to express the data, and the analyses were performed using t‐tests for 2‐group comparisons and two‐way ANOVA for comparisons of 3 groups or more. A *p* value of < 0.05 was considered to indicate statistical significance (^*^, *p* < 0.05; ^**^, *p* < 0.01; ^***^, *p* < 0.001).

### Ethics Approval

The present study was approved by the Ethics Committee of Fudan University Shanghai Cancer Center (050432‐4‐2108*) and the Third Affiliated Hospital of Kunming Medical University (KYCS2022004).

## Conflict of Interest

The authors declare no conflict of interest.

## Author Contributions

B.C., Y.W., and H.H.Y. contributed equally to this work.: B.S.C, Y.L.L., Y.S., and S.W. performed conception and design. B.C., Y.S., Y.L.L., and Z.L.M. performed the development of the methodology. B.C., Y.S., B.F.F., and J.M. performed analysis and interpretation of data (e.g., statistical analysis, biostatistics, computational analysis). B.C., Y.S., W.P., W.M., and S.W. wrote, review, and/or revision of the manuscript. Y.W., H.H.Y., S.C., YlQ.X., and Y.K.Z. performed clinical sample collection. S.W., Y.S., X.F.Z., and Z.X.H. performed administrative, technical, or material support (i.e., reporting or organizing data, constructing databases).

## Supporting information

Supporting Information

Supporting Information

Supporting Information

## Data Availability

The data that support the findings of this study are available from the corresponding author upon reasonable request.
