# Supplementary material for: Tau Aggregation‐Dependent Lipid Peroxide Accumulation Driven by the hsa_circ_0001546/14‐3‐3/CAMK2D/Tau Complex Inhibits Epithelial Ovarian Cancer Peritoneal Metastasis
Source: Adv Sci (Weinh). 2024 Apr 18;11(23):2310134. doi: 10.1002/advs.202310134 (PMC11186043; doi:10.1002/advs.202310134)
Supplement: Supplementary file 1 — Supporting Information [file ADVS-11-2310134-s003.pdf]

ADVANCED SCIENCE
Open Access

Supporting Information

for *Adv. Sci.*, DOI 10.1002/advs.202310134

Tau Aggregation-Dependent Lipid Peroxide Accumulation Driven by the hsa_circ_0001546/14-3-3/CAMK2D/Tau Complex Inhibits Epithelial Ovarian Cancer Peritoneal Metastasis

BinShu Chai, Yong Wu, HengHui Yang, BiaoFeng Fan, SiYu Cao, XiaoFei Zhang, YaQing Xie, ZhiXiang Hu, ZhongLiang Ma, YunKui Zhang, Wei Pan, Wei Meng, Jiao Meng, WenJuan Tian, JiaLi Zhang, YanLi Li, Yang Shao* and ShaoJia Wang**

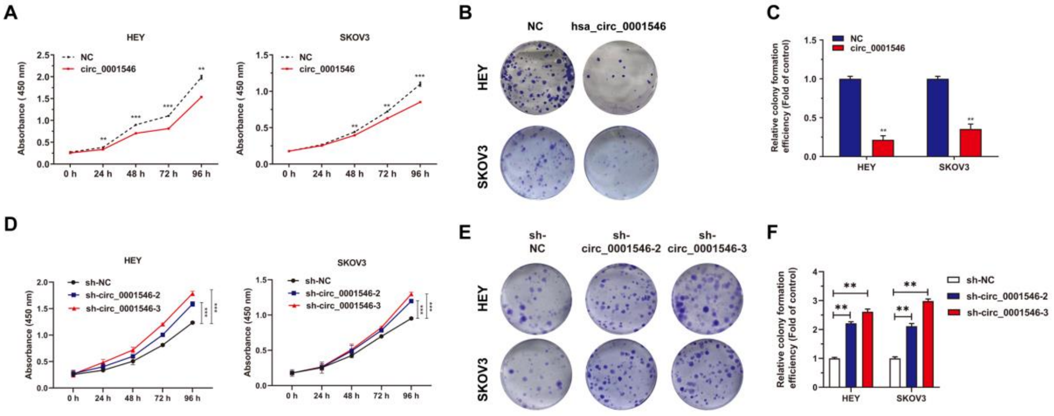

Supplementary Fig.S1 hsa_circ_0001546 suppresses EOC proliferation *in vitro*.

A. CCK-8 assay for hsa_circ_0001546 overexpressing HEY and SKOV3 cells.

B-C. Colony formation assays for hsa_circ_0001546 overexpressing HEY and SKOV3 cells.

D. CCK-8 assay for hsa_circ_0001546 knockdown HEY and SKOV3 cells.

E-F. Colony formation assays for hsa_circ_0001546 knockdown HEY and SKOV3 cells.

**$P < 0.01$, ***$P < 0.001$. Three independent experiments were performed.

A

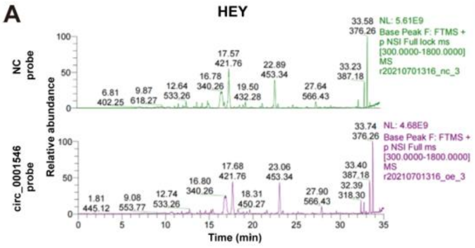

B

Protein/Gene name	NC probe	circ_0001546 probe	Fold change
14-3-3 protein beta/alpha(YWHAB)	6200675.7	20335595.9	3.3
14-3-3 protein eta(YWHAH)	6335185.0	26919912.7	4.2
14-3-3 protein theta(YWHAQ)	847232.1	4908744.7	5.8
14-3-3 protein zeta/delta(YWHAZ)	4984135.4	20712462.9	4.2

C

Protein region	RNA region	Interaction Propensity	Discriminative Power	Normalized Score
26-77	242-293	9.47	28	3.07
146-197	242-293	7.84	24	2.61
76-127	242-293	7.69	24	2.57
26-77	251-302	7.51	24	2.52
26-77	267-318	6.98	22	2.38
151-202	242-293	6.65	22	2.28
101-152	242-293	6.61	22	2.27
146-197	251-302	6.02	22	2.11
76-127	251-302	5.73	22	2.03
146-197	267-318	5.41	22	1.94
71-122	242-293	5.39	22	1.94
51-102	226-277	5.19	22	1.88
126-177	226-277	5.18	22	1.88

D

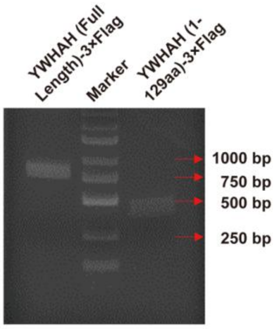

Supplementary Fig.S2 The mass spectrometry analysis of the interaction between hsa_circ_0001546 and 14-3-3 proteins in EOC cells.

A. The mass spectra of hsa_circ_0001546 RNA pull-down assay.

B. The analysis summarizes the combination of hsa_circ_0001546 and 14-3-3 proteins.

C. Potential binding sites of hsa_circ_0001546 and YWHAH predicted by catRAPID database, including 26-77aa, 76-127aa, 151-202aa and 51-102aa regions.

D. The constructed plasmids including YWHAH (Full Length)-3×Flag and YWHAH (1-129aa)-3×Flag were verified by agarose gel electrophoresis.

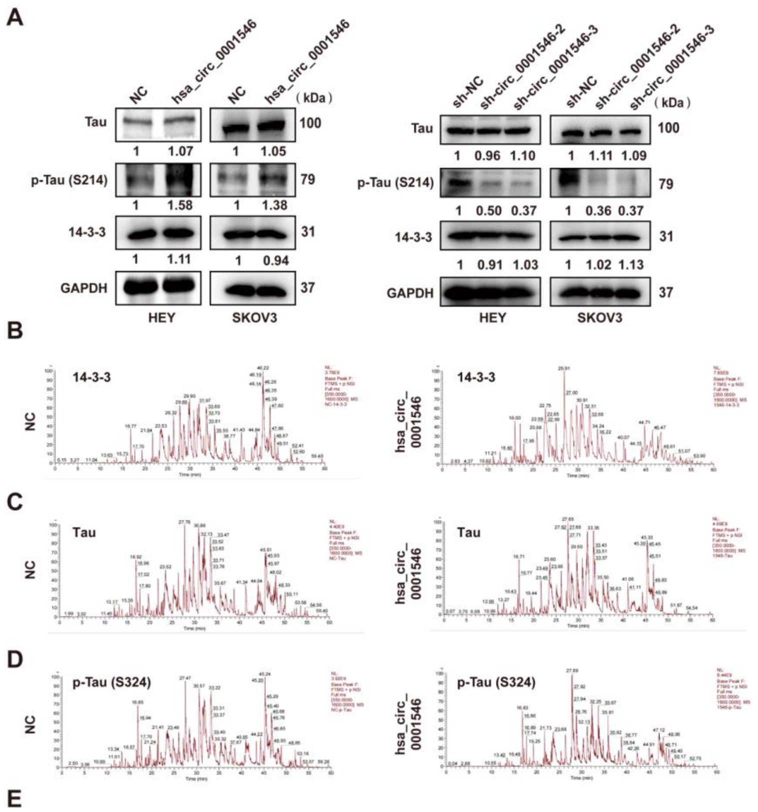

E

HEY	Gene name	Coverage [%]	Peptides	PSMs	Protein Unique Peptides	Unique Peptides	Score
NC	CAMK2D	10	3	5	2	2	15.88
circ_0001546	CAMK2D	21	8	11	3	4	31.87

Supplementary Fig.S3 The mass spectrometry analysis of the binding between hsa_circ_0001546, 14-3-3 proteins, CAMK2D and p-Tau in EOC cells.

A. The protein levels of Tau, p-Tau (S214) and 14-3-3 were detected in hsa_circ_0001546 stable-overexpressing and stable-down-expressing cells.

B. The mass spectra of Co-IP assays with 14-3-3 primary antibody.

C. The mass spectra of Co-IP assays with Tau primary antibody.

D. The mass spectra of Co-IP assays with p-Tau (S324) primary antibody.

E. The analysis summarizes the binding of protein kinase CAMK2D and p-Tau (S324).

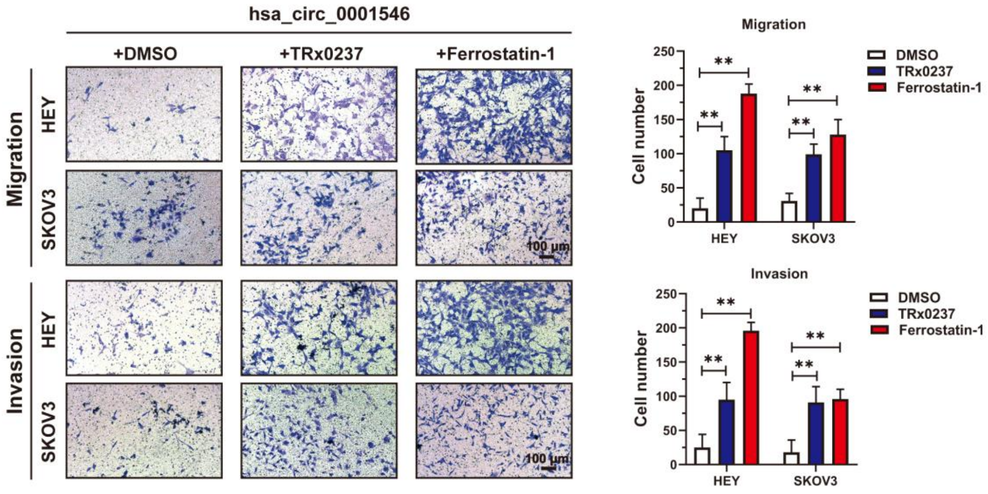

Supplementary Fig.S4 Ferrostatin-1 and TRx0237 rescue the inhibitory effect of hsa_circ_0001546 on EOC cell migration and invasion. Scale bar = 100 μm.

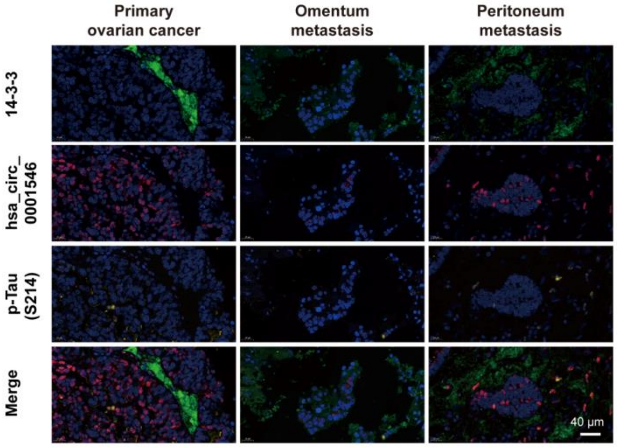

Supplementary Fig.S5 Subcellular co-localization and expression of hsa_circ_0001546 (Red), 14-3-3 (Green) and p-Tau (S214, Yellow) detected by IF and FISH assays in normal ovarian tissues and paired primary ovarian cancer, omentum metastasis and peritoneum metastasis tissues. Scale bar = 40 μm.

Supplementary Table S1

Details of total EOC clinical samples are listed.

Supplementary Table S2

Sequences of all shRNA, primers and probes are listed.
