# Supplementary material for: Tau Aggregation‐Dependent Lipid Peroxide Accumulation Driven by the hsa_circ_0001546/14‐3‐3/CAMK2D/Tau Complex Inhibits Epithelial Ovarian Cancer Peritoneal Metastasis
Source: Adv Sci (Weinh). 2024 Apr 18;11(23):2310134. doi: 10.1002/advs.202310134 (PMC11186043; doi:10.1002/advs.202310134)
Supplement: Supplementary file 2 — Supporting Information [file ADVS-11-2310134-s002.pdf]

ADVANCED SCIENCE
Open Access

Supporting Information

for *Adv. Sci.*, DOI 10.1002/advs.202310134

Tau Aggregation-Dependent Lipid Peroxide Accumulation Driven by the hsa_circ_0001546/14-3-3/CAMK2D/Tau Complex Inhibits Epithelial Ovarian Cancer Peritoneal Metastasis

BinShu Chai, Yong Wu, HengHui Yang, BiaoFeng Fan, SiYu Cao, XiaoFei Zhang, YaQing Xie, ZhiXiang Hu, ZhongLiang Ma, YunKui Zhang, Wei Pan, Wei Meng, Jiao Meng, WenJuan Tian, JiaLi Zhang, YanLi Li, Yang Shao* and ShaoJia Wang**

Table S1 Descriptive characteristics of EOC samples

Characteristic	Epithelial Ovarian Cancer (n=68)
**Median age (range)**	55 (23-77)
**Average age (range)**	54 (23-77)
**Gender**	
Male	0 (0%)
Female	68 (100%)
**Stage, n (%)**	
Ⅰ	0 (0%)
Ⅱ	0 (0%)
Ⅲ	22 (32.35%)
Ⅳ	46 (67.65%)
**Pathological characteristics, n (%)**	
Metastasis	27 (39.71%)
Lymphatic metastasis	27 (39.71%)
Lympho-vascular invasion (LVI)	27 (39.71%)
Neural invasion	7 (10.29%)
Recurrence	30 (44.12%)
