# Supplementary material for: Tau Aggregation‐Dependent Lipid Peroxide Accumulation Driven by the hsa_circ_0001546/14‐3‐3/CAMK2D/Tau Complex Inhibits Epithelial Ovarian Cancer Peritoneal Metastasis
Source: Adv Sci (Weinh). 2024 Apr 18;11(23):2310134. doi: 10.1002/advs.202310134 (PMC11186043; doi:10.1002/advs.202310134)
Supplement: Supplementary file 3 — Supporting Information [file ADVS-11-2310134-s001.pdf]

ADVANCED SCIENCE
Open Access

Supporting Information

for *Adv. Sci.*, DOI 10.1002/advs.202310134

Tau Aggregation-Dependent Lipid Peroxide Accumulation Driven by the hsa_circ_0001546/14-3-3/CAMK2D/Tau Complex Inhibits Epithelial Ovarian Cancer Peritoneal Metastasis

BinShu Chai, Yong Wu, HengHui Yang, BiaoFeng Fan, SiYu Cao, XiaoFei Zhang, YaQing Xie, ZhiXiang Hu, ZhongLiang Ma, YunKui Zhang, Wei Pan, Wei Meng, Jiao Meng, WenJuan Tian, JiaLi Zhang, YanLi Li, Yang Shao* and ShaoJia Wang**

Table S2 Primer & shRNA& Probe sequences

Primer & shRNA & Probe Name	Primer & shRNA & Probe Sequences（5'-3'）
18s RNA qRT-PCR primer	F: AGGAATTCCCAGTAAGTGCG<br>R: GCCTCACTAAACCATCCAA
hsa_circ_0001546 qRT-PCR primer	F: CAAGTATGTAGCAGCTGATGTT<br>R: TATCCTGAATAGGGAGAACTTT
hsa_circ_0001546 divergent primer	F: TGGAATCCCTGGTCCCAGTGAA<br>R: TTCTTGGCTGGCTCACAGTTTC
hsa_circ_0001546 convergent primer	F: GAAAGAAGAGATACGGACCT<br>R: TTTATTTCACTTTCTTGGGA
sh-circ_0001546-1	F: CCGGGTATGTAGCAGCTGATGTTCTCTCGAGAGAACATCAGCTGCTACATACTTTTTG<br>R: AATTCAAAAAGTATGTAGCAGCTGATGTTCTCTCGAGAGAACATCAGCTGCTACATAC
sh-circ_0001546-2	F: CCGGGTAGCAGCTGATGTTCTAATCCTCGAGGATTAGAACATCAGCTGCTACTTTTTG<br>R: AATTCAAAAAGTAGCAGCTGATGTTCTAATCCTCGAGGATTAGAACATCAGCTGCTAC
sh-circ_0001546-3	F: CCGGGCAGCTGATGTTCTAATCATGCTCGAGCATGATTAGAACATCAGCTGCTTTTTG<br>R: AATTCAAAAAGCAGCTGATGTTCTAATCATGCTCGAGCATGATTAGAACATCAGCTGC
hsa_circ_0001546 RNA pull-down probe	AUUAGAACAUCAGCUGCUACAUACU
hsa_circ_0001546 FISH probe	TAGAACATCAGCTGCTACATACTTGACTT
